# Development and Validation of a Comprehensive Well-Being Scale for People in the University Environment (Pitt Wellness Scale) Using a Crowdsourcing Approach: Cross-Sectional Study

**DOI:** 10.2196/15075

**Published:** 2020-04-29

**Authors:** Leming Zhou, Bambang Parmanto

**Affiliations:** 1 Department of Health Information Management University of Pittsburgh Pittsburgh, PA United States

**Keywords:** crowdsourcing, questionnaire design, university

## Abstract

**Background:**

Well-being has multiple domains, and these domains are unique to the population being examined. Therefore, to precisely assess the well-being of a population, a scale specifically designed for that population is needed.

**Objective:**

The goal of this study was to design and validate a comprehensive well-being scale for people in a university environment, including students, faculty, and staff.

**Methods:**

A crowdsourcing approach was used to determine relevant domains for the comprehensive well-being scale in this population and identify specific questions to include in each domain. A web-based questionnaire (Q1) was used to collect opinions from a group of university students, faculty, and staff about the domains and subdomains of the scale. A draft of a new well-being scale (Q2) was created in response to the information collected via Q1, and a second group of study participants was invited to evaluate the relevance and clarity of each statement. A newly created well-being scale (Q3) was then used by a third group of university students, faculty, and staff. A psychometric analysis was performed on the data collected via Q3 to determine the validity and reliability of the well-being scale.

**Results:**

In the first step, a group of 518 university community members (students, faculty, and staff) indicated the domains and subdomains that they desired to have in a comprehensive well-being scale. In the second step, a second group of 167 students, faculty, and staff evaluated the relevance and clarity of the proposed statements in each domain. In the third step, a third group of 546 students, faculty, and staff provided their responses to the new well-being scale (Pitt Wellness Scale). The psychometric analysis indicated that the reliability of the well-being scale was high.

**Conclusions:**

Using a crowdsourcing approach, we successfully created a comprehensive and highly reliable well-being scale for people in the university environment. Our new Pitt Wellness Scale may be used to measure the well-being of people in the university environment.

## Introduction

### Background

Well-being is “a good or satisfactory condition of existence; a state characterized by health, happiness, and prosperity” [1]. Well-being is commonly assessed using well-being scales.

Few well-being scales, such as the Patient-Reported Outcomes Measurement Information System (PROMIS), 36-Item Short-Form Health Survey (SF-36), and World Health Organization Quality of Life scale, have been designed for the general population [2-4]. However, while these generic well-being scales are useful for large-scale assessments and for obtaining an overall impression of a population, they may not be able to accurately reflect the well-being situation of a particular population. Therefore, a population-specific well-being scale is needed to precisely assess the well-being of the target population.

Many well-being and quality of life scales have been created for different purposes and for different target populations [5-8]. Some well-being and quality of life scales were specifically created for people with particular diseases, such as depression, stroke, and cancer [9-12]. Others were created for particular populations, such as children and adults [13-15]. These specific scales are very useful for assessing the well-being of the target population, but they are not appropriate for other populations.

People in the university environment (students, faculty, and staff) can be considered a specific population. The activities conducted by people in the university environment and the relationships among them are different from those of people in government offices, companies, hospitals, and even elementary and secondary education schools. People in the university environment are focused on higher education, research, and career development. At the same time, universities are not ivory towers. People in the university environment (students, faculty, and staff) are not isolated from the world, and they have a life outside of teaching, research, service, and learning. Like other people, they experience various problems in real life, such as physical disease, mental problems, financial pressure, and problems related to handling relationships with difficult people around them.

In recent years, several important discussions have arisen regarding college students’ health issues, faculty and student relationships, and university employees’ job satisfaction [16-22]. These discussions often only focused on a specific issue, such as physical health [23,24], harmful lifestyle [25,26], and mental health [16,27,28]. In many cases, however, these issues are intertwined; for instance, mental illness or abnormal behavior may be triggered by heavy academic workload, severe financial pressure, and poor relationships with others [17,29,30]. Therefore, it is necessary to use tools to perform a *comprehensive* well-being assessment in order to provide a foundation for well-being improvement interventions. At present, there is no well-being scale specifically designed for people in the university environment (students, faculty, and staff).

Well-being is a higher order construct, and thus, it includes multiple lower order constructs or domains [6,31,32]. The commonly covered domains in well-being scales are physical, emotional (or mental), social (or relational), spiritual, and financial (or socioeconomic) [6,32]. Some well-being scales also cover occupational, environmental, and intellectual domains [6,32]. These domains may have one or multiple subdomains. For instance, the physical domain may include subdomains, such as physical health, daily living activities, pain, and sleep; the social domain may include participation, friends, and other relationships; and the mental domain may include happiness, depression, stress coping skills, and communication.

To conduct a comprehensive well-being assessment for people in the university environment, we need a scale with multiple domains [6,31,32]. There are a number of domain-specific scales, such as the social interaction anxiety scale (SIAS) for social interaction anxiety, and a number of PROMIS scales for pain [33], smoking [34], and depression [35]. However, we cannot simply use a combination of multiple existing domain-specific scales to build our well-being scale for two major reasons. First, the comprehensive well-being of different populations needs to be measured using different sets of domains. The combination of these domains can only be determined by the target population. Second, the wording of some statements and subdomains for existing domain-specific scales may not be applicable for our target population, as many scales were created with certain populations in mind, such as elderly people, healthy young professionals, people with cancer, and people who play a particular role (eg, caregiver). Hence, in this project, it was necessary to first identify the domains and related subdomains of a comprehensive well-being scale relevant for people in the university environment and then create statements that use language appropriate to this population for each subdomain.

The typical scale development approach used by researchers involves conducting a literature review, drafting a scale for a small group of experts to review, and then releasing the new scale to a group of recruited study participants to collect responses. In this typical approach, a sample of the target population is only involved in the last stage of scale development. This is a shortcoming in that study participants are simply asked to provide responses to the statements in the scale, and thus, any domains or subdomains that researchers may have missed in the draft of the scale will not be brought to the researchers’ attention. Crowdsourcing is one way to overcome this issue.

In recent years, crowdsourcing has been used to collect ideas from a crowd [36-38]. The benefit of crowdsourcing is that the collected wisdom of the crowd can be identified by using feedback obtained from a large pool of the target population [39-45]. On the other hand, most people in the crowd do not have formal training in research or scale development; therefore, the information from the crowd cannot be solely depended on to create a new scale. We have adopted what we believe is a better strategy. It involves combining these two approaches in the development of our new comprehensive well-being scale in order to retain the advantages of these two methods while avoiding their limitations.

More specifically, in this combined approach, information from the literature was used to guide the development of the new scale, and the crowd participated in all stages of the scale development and evaluation (domain and subdomain determination, statement relevance and clarity evaluation, and response to statements) to fully reflect their ideas in the new scale.

Before describing the objective of this study, we present the definitions of several commonly referred well-being domains below. They have been adopted from a previous study [6].

*Physical wellness* refers to “the quality and performance of bodily functioning.” *Emotional wellness* reflects “the psychological, cognitive, and emotional quality of a person’s life.” *Social wellness* is about “how well an individual is connected to others in their local and wider social community.” *Spiritual wellness* is about “meaning, a connection to something greater than oneself, and in some cases, faith in a higher power.” *Financial wellness* refers to “an individual’s financial management skills and financial security.” *Occupational wellness* indicates “an individual’s career development opportunities and job satisfaction.” *Intellectual wellness* refers to “an individual’s ability to handle tasks in daily life and on the job, and their self-assessment of their performance.”

### Objective

The goal of this study was to create a comprehensive well-being scale for people in the university environment, using a combined approach (traditional scale development method and crowdsourcing). The study also sought to achieve acceptable reliability of the new scale to demonstrate the benefits of using this combined scale development approach.

## Methods

### User-Centered Approach for Scale Development and Evaluation

**Figure 1:**
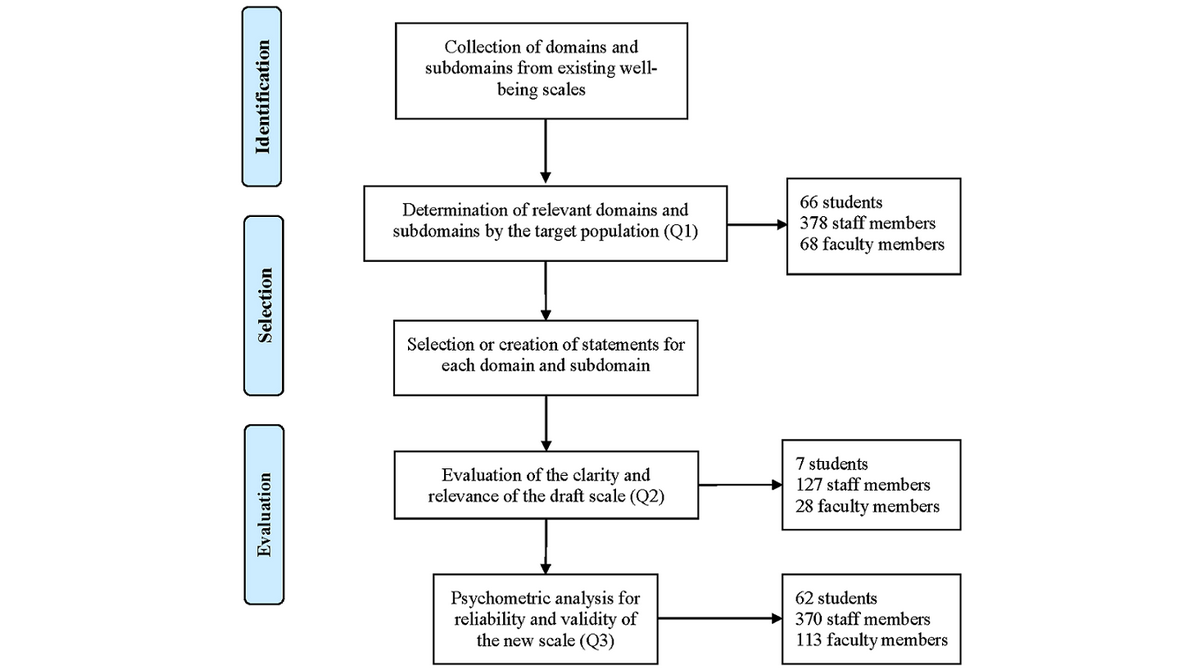
Flowchart of the design and evaluation of the well-being questionnaire.

User-centered design is the process of developing a tool from the perspective of how it will be understood and used by users [46]. Therefore, in a user-centered design, the target users of a tool are actively involved in all stages of the product development. In scale development, this includes domain and subdomain identification, statement selection in terms of validity, and scale evaluation in terms of validity and reliability. These are the general steps we took in this study (Figure 1). The details of each step are provided in the following sections.

### Study Procedure

In this study, well-being scales and their corresponding domains and subdomains were collected from several recent review studies on well-being and quality of life scales [5-8,31,47]. A web-based questionnaire (Q1) was created to collect opinions from people in the university environment (students, faculty, and staff) on these domains and subdomains for their own well-being assessment. The obtained results were used to guide the creation of the first draft of a new comprehensive well-being scale. This draft was provided to people in the university environment via another web-based questionnaire (Q2) to obtain their evaluation of the relevance and clarity of each statement. A revised well-being scale based on input from the draft was then released to people in the university environment in order to collect responses to its statements via a third web-based questionnaire (Q3). All of the study participants were encouraged to provide comments and suggestions on each statement in these three questionnaires and on the new scale. A psychometric analysis was performed to evaluate the reliability and validity of the new comprehensive well-being scale. This study protocol was approved by the Human Research Protection Office at the University of Pittsburgh (Pitt). The details of each step are provided below.

#### Step 1: Collection of Domains and Subdomains From Existing Well-Being Scales

Well-being and quality of life scales from six recent review studies were collected [5-8,31,47]. The domains and subdomains of these scales were compiled. The statements of these scales were also compiled.

#### Step 2: Determination of Relevant Domains and Subdomains According to the Target Population

It is known that domains and their subdomains vary widely in different well-being scales [6]. In this study, to get an idea of which domains and subdomains are most valuable in assessing the well-being of a university population, study participants were asked to fill out a web-based questionnaire (Q1) with a list of domains and subdomains. Study participants were asked to provide their opinions on the relevance of these domains and subdomains for their own well-being assessment.

#### Step 3: Evaluation of the Clarity and Relevance of a Draft Scale

Statements were selected from existing well-being and quality of life scales for domains and subdomains identified as relevant to the university population in step 2. For domains having only few already existing statements, such as the intellectual domain, new statements were created. The collection of these statements in each domain and subdomain formed the first draft of the new well-being scale. We had multiple rounds of discussions on the clarity of each statement and relevance of each statement to the corresponding domain and subdomain. A final draft of 77 statements was provided via a web-based questionnaire (Q2) to people in the university environment for evaluation of the relevance and clarity of each statement. These study participants evaluated the relevance and clarity of each statement for use in a comprehensive well-being scale, using a scale from 1 to 4, where 1 meant no relevance or clarity and 4 meant high relevance or clarity. In response to these evaluations, if the average relevance of a statement was lower than 2.5, it was removed from the scale. If the clarity of a statement was rated 1 or 2, the wording of the statement was adjusted. We had multiple face-to-face meetings to discuss the rating and wording of statements for finalizing the draft scale.

#### Step 4: Questionnaire Study and Psychometric Analysis for Reliability and Validity

After we agreed on the content validity of the statements in the new well-being scale, the scale was released to people in the university environment via a web-based questionnaire (Q3) in order to collect study participants’ answers to the statements in the questionnaire. The obtained data were used to evaluate the reliability and validity of the new scale. The details of the data analysis are presented in a later section.

### Participant Recruitment

In this study, the study participants were current Pitt students, staff, and faculty who were randomly selected by a bulk email system. Former students, staff, and faculty were excluded from the study, because they might have been working in a different environment for a long time and hence their opinion might not reflect the actual well-being of someone currently in the university environment.

To recruit study participants, emails about the purpose of the study and links to the corresponding questionnaires (Q1, Q2, and Q3) were randomly distributed to approximately 2000 current students, staff, and faculty at Pitt via a bulk email system (Read Green) at different time points for each questionnaire study. This Pitt bulk email system has all the email addresses of current Pitt students, staff, and faculty. According to the Pitt Fact Book 2019, the total number of email addresses included in the bulk email system was close to 50,000 (one per person). When we requested to make an announcement via this bulk email system, we were required to indicate the number of people and the categories of the university members. The number of email addresses requested was directly linked to the charge of the email distribution service. The bulk email system randomly picked email addresses from each indicated category (students, staff, and faculty) among the 50,000 email addresses, for a total of 2000 email addresses, and sent out the announcement. Since the three announcements were made at three different time points (separated by approximately 1 month), the 2000 email addresses in Q1, Q2, and Q3 could be completely different or have very limited overlap. In other words, one Pitt student, staff member, or faculty member might have received one, two (unlikely), or three (very unlikely) email announcements because of the randomness of the email selection.

To participate in the study, students, faculty, and staff who received the email message could click on the link to the questionnaire given in the email and provide their responses on the web-based Qualtrics system. The purpose of the study was also described at the beginning of each questionnaire. Study participation was voluntary, and participants could stop participating in the study at any time. They could also request that their entered data be removed in the comments section of the questionnaires.

Participants were asked to provide some basic demographic information, such as age, gender, race, education, and role at Pitt, before they responded to any other statements in the questionnaires. Their responses were stored anonymously, since they were not required to provide their name, department, or job. The Internet Protocol (IP) addresses of their computers were hidden to the investigators.

### Data Analysis

A descriptive analysis was performed on the collected data to understand the demographic characteristics of the study participants and the overall results from the data-collection questionnaires, such as the mean and SD values of individual statements. The comments and suggestions collected by open-ended questions in the three web-based questionnaires were summarized briefly.

Cronbach alpha was calculated for each domain of the scale and the entire well-being scale to evaluate the reliability of the scale. Cronbach alpha is a commonly used measurement of internal consistency for questionnaires. For research and exploratory studies, Cronbach alpha values from .7 to .8 are considered acceptable, whereas a value around .9 is considered excellent [48].

Exploratory factor analysis and confirmatory factor analysis were performed to determine and verify the constructs of the new well-being scale. In the exploratory factor analysis, the extraction method was principal component analysis and the rotation method was Oblimin with Kaiser Normalization [49,50]. The factor loadings obtained in the exploratory factor analysis were used to determine whether each statement should be included in the well-being scale and in one specific domain. Here, 0.32 was used as the guiding value for the evaluation [51]. However, in certain cases, we overruled this value and chose to keep a statement in the scale, even if the factor loadings were smaller than 0.32 or multiple factor loadings were greater than 0.32, using judgement skills gained from our extensive experience in scale development and the opinions of the target population obtained in the first web-based questionnaire study (Q1). R package LAVAAN 0.5 (Yves Rosseel et al, Belgium) was used for the confirmatory factor analysis. The estimator was maximum likelihood. A two-layer multi-factor model was used in this analysis. The domains were latent variables, and their items were the observables. All the domains together were used to measure overall well-being. All the statistical analyses were performed using R 3.3 (The R Foundation, Vienna, Austria) and IBM SPSS version 24 (IBM Corp, Armonk, New York, USA).

## Results

### Identified Scales

In total, 165 well-being and quality of life scales were collected from previous review studies. The total number of statements in these scales was approximately 4700. We cannot provide an exact number for the total because some scales have multiple versions with different numbers of items. A few hundred domains were covered in these scales; however, most of them were only mentioned in one or a few scales. We chose the following seven frequently covered domains for well-being assessment: physical, emotional, social, spiritual, financial, occupational, and intellectual. Their subdomains, which were found in multiple scales, were identified as well. These domains and subdomains were listed in the web-based questionnaire (Q1) so that the study participants could make selections. The definitions of the seven domains were given in the questionnaire so that every participant would know the meaning of each domain. The subdomains were more specific, and thus, no definitions were provided for them.

### Domains and Subdomains

After Q1 was distributed to approximately 2000 students, faculty, and staff via email, 518 of the recipients chose to answer the questionnaire. Their mean age was 41.6 years (SD 13.4). Further details on their demographics are summarized in Table 1.

The responses from these 518 study participants in the first questionnaire study (Q1) are summarized in Table 2. The responses were organized into categories. These categories were then broken down into domains and subdomains. In Table 2, the information is listed in the order of importance for inclusion in a comprehensive well-being assessment, as indicated by the participants’ responses.

**Table 1:** Demographic information of the 518 study participants in the first questionnaire study.

Characteristic		Value, n (%)
**Role**		
	Student	66 (12.7)
	Staff	378 (73.0)
	Faculty	68 (13.1)
**Gender**		
	Male	107 (20.7)
	Female	405 (78.2)
	Undeclared	6 (1.1)
**Race**		
	African American	21 (4.1)
	White American	455 (87.8)
	Asian American	24 (4.6)
	Other (mixed race, Native American, or Hispanic)	13 (2.6)
**Education**		
	High school or lower	12 (2.3)
	Some college credits, no degree	40 (7.7)
	Associate degree	20 (3.9)
	Bachelor’s degree	194 (37.5)
	Master’s degree	170 (32.8)
	Professional degree	13 (2.5)
	Doctoral degree	66 (12.7)

**Table 2:** Summary of answers from the study participants in the first questionnaire study (N=518).

Question				Value, n (%)
**Which of the following domains are important for a comprehensive well-being assessment?**				
	Physical wellness		505 (97.5)	
	Emotional wellness		493 (95.2)	
	Financial wellness		374 (72.2)	
	Social wellness		338 (65.3)	
	Occupational wellness		329 (63.5)	
	Spiritual wellness		245 (47.3)	
	Intellectual wellness		236 (45.6)	
	Other (eg, environmental wellness)		21 (4.1)	
**Please indicate the subdomains for each domain, which you believe are important for your comprehensive well-being assessment.**				
	**Physical wellness**			
		Physical activity	483 (93.2)	
		Nutrition	480 (92.7)	
		Sleep	468 (90.3)	
		Overall health	408 (78.8)	
		Chronic disease	216 (41.7)	
		Medication dependence	178 (34.4)	
		Appetite	114 (22.0)	
		Other	25 (4.8)	
	**Emotional wellness**			
		Stress	416 (80.3)	
		Positive attitude	386 (74.5)	
		Anxiety	369 (71.2)	
		Resilience	306 (59.1)	
		Depression or bipolar disorder	300 (57.9)	
		Traumatic events	225 (43.4)	
		Posttraumatic stress disorder	200 (38.6)	
		Negative attitude	152 (29.3)	
		Other	36 (6.9)	
	**Social wellness**			
		Relationship with family, friends, and colleagues	488 (94.2)	
		Connection with others	425 (82.0)	
		Social participation	341 (65.8)	
		Smoking, alcohol, and drug use	117 (22.6)	
		Other	16 (3.1)	
	**Financial wellness**			
		Preparedness for short-term and long-term financial emergency	434 (83.8)	
		Skills for financial management	427 (82.4)	
		Income level	274 (52.9)	
		Other	35 (6.8)	
	**Spiritual wellness**			
		Purpose of life	311 (60.0)	
		Satisfaction with the current belief system	266 (51.4)	
		View of the world	259 (50.0)	
		Meaning of life	207 (40.0)	
		Meditation	181 (34.9)	
		Spiritual activities	160 (30.9)	
		Religion	153 (29.5)	
		Other	26 (5.0)	
	**Occupational wellness**			
		Job satisfaction	486 (93.8)	
		Job security	405 (78.2)	
		Career development opportunities	362 (69.9)	
		Job stress	326 (62.9)	
		Job performance	234 (45.2)	
		Career ambition	189 (36.5)	
		Workaholic (job and life balance)	85 (16.4)	
		Other	34 (6.6)	
	**Intellectual wellness**			
		Capacity for thinking and acquiring knowledge	455 (87.8)	
		View on life-long learning (burden, part of life, or enjoy)	386 (74.5)	
		Informal education experience	254 (49.0)	
		Formal education experience	230 (44.4)	
		Other	19 (3.7)	
**How many questions are reasonable for assessing each domain of your well-being?**				
	10		231 (44.6)	
	5		111 (21.4)	
	15		90 (17.4)	
	20		71 (13.7)	

One additional category, environmental wellness, was added by 21 (4.05%) participants as an aspect of wellness that is important, indicating that these individuals live a lifestyle that is mindful of their surroundings. Study participants also identified many more subdomains in each domain than were in the original questionnaire. Multimedia Appendix 1 provides a list of additional subdomains mentioned by some study participants. Some study participants also made general comments on the well-being scale creation activity itself.

Great idea. You cannot improve something if you don’t track it first.

Participant #341 in Q1

Make sure it is available to all doctors that a person will visit so they can use it as a baseline.

Participant #355 in Q1

### Relevance and Clarity of the Proposed Statements

A draft of a well-being scale with 77 statements, which included those domains and subdomains designated as important by the participants in the Q1 study, was created. Seven domains were included, and on average, there were 10 statements in each domain (also according to the responses of many participants in the Q1 study). Most of these statements were selected or modified from existing well-being and quality of life scales, except for those in the financial and intellectual domains, which were mainly written by us, as existing scales did not include many such statements.

This draft was randomly distributed to another 2000 university members to obtain their feedback (as members of the target population) on the relevance and clarity of each statement via the second web-based questionnaire (Q2). In total, 167 participants responded, and of these, 143 (85.6%) provided their ratings on the relevance and clarity of all 77 statements and the other 24 provided their ratings for at least one-third of the statements in the draft scale. The mean age of this group of participants was 44.0 years (SD 12.99). Among the 167 participants, there were 127 staff members (76.0%), 28 faculty members (16.8%), and 7 students (4.2%). Most participants were female (132/167, 79.0%). There were 30 (18.0%) male participants and 5 (3.0%) who did not indicate gender. As indicated in the Methods section, statements with an average rating of relevance lower than 2.5 were directly removed from the draft scale. The wording of statements was adjusted if participants were confused by the statements (clarity rating was 1 or 2). A few statements were removed because they were highly personal and study participants expressed a strong objection to them (eg, a statement about sexual activity). At the end of this step, the updated well-being scale had 47 statements in total.

### Evaluation of the New Well-Being Scale

The updated well-being scale (named the Pitt Wellness Scale), several demographic questions, and few open-ended questions for comments and suggestions were combined to create the third web-based questionnaire (Q3). The link to Q3 was again randomly distributed to approximately 2000 university community members. In total, 671 individuals clicked on the link to this questionnaire, and 546 of them provided responses to all of the statements in the new well-being scale. This new scale was evaluated using the responses from these 546 participants. The mean duration of response to all the statements was 535.32 seconds (minimum 117, maximum 14,794, SD 1187.33; less than 10 minutes), which is an acceptable length of time for most people. The mean age of the participants in the Q3 study was 43.7 years (SD 13.54). Further details on their demographics are provided in Table 3.

Descriptive statistics of responses were calculated, and a reliability test of the scale was performed. For most statements, response options ranged on a scale from 1 (strongly agree) to 7 (strongly disagree). Eight statements (self-assessed level of wellness for each domain and overall wellness) had options ranging on a scale from 1 (excellent) to 5 (terrible). The options for the level of pain statement ranged from 0 (no pain) to 10 (most severe pain ever). After the reliability analysis, three statements were removed to improve the reliability of the well-being scale. Therefore, the final version of the new well-being scale included 44 statements. The overall Cronbach alpha of the 44-item scale was .933. Table 4 presents the descriptive statistics, Cronbach alpha value of each domain, and number of items in each domain.

An exploratory factor analysis was performed on the responses from the 546 study participants, assuming there were seven factors in this scale. The obtained pattern matrix is shown in Table 5. Here, rotated factor loadings greater than 0.32 are shown. Two statements (WO and P6) with factor loadings less than 0.32 are also shown. WO is for overall well-being and therefore does not belong to any domain. P6 is about appetite, which is highly relevant to both physical health and mental health. Therefore, although the highest factor loading for P6 was 0.301, we still chose to keep this statement in the scale. The reliability evaluation indicated that both physical and mental domains had higher reliability when P6 was in the physical domain (Cronbach alpha, .705 [without P6] vs .714 [with P6] for the physical domain and .857 [with P6] vs .860 [without P6] for the mental domain). Therefore, we kept P6 in the physical domain.

**Table 3:** Demographic information of the 546 study participants who provided responses to all the statements in the new well-being scale.

Characteristic		Value, n (%)
**Role**		
	Student	62 (11.4)
	Faculty	113 (20.7)
	Staff	370 (67.8)
	Undeclared	1 (0.2)
**Gender**		
	Male	128 (23.4)
	Female	411 (75.3)
	Undeclared	7 (1.3)
**Race**		
	African American	16 (2.9)
	White American	496 (90.8)
	Asian American	22 (4.0)
	Other	12 (2.2)
**Education**		
	High school or lower	4 (0.7)
	Some college credits, no degree	24 (4.4)
	Some technical training, no degree	11 (2.0)
	Associate degree	18 (3.3)
	Bachelor’s degree	194 (35.5)
	Master’s degree	163 (29.9)
	Professional degree	22 (4.0)
	Doctoral degree	110 (20.1)
**Marital status**		
	Single	137 (25.1)
	Married or long-term committed relationship	369 (67.6)
	Divorced or separated	34 (6.2)
	Widowed	6 (1.1)
**Household income**		
	≤US $10,000	7 (1.3)
	US $10,001-25,000	14 (2.6)
	US $25,001-50,000	114 (20.9)
	US $50,001-75,000	91 (16.7)
	US $75,001-100,000	84 (15.4)
	US $100,001-125,000	74 (13.6)
	>US $125,000	143 (26.2)
	Declined to answer	19 (3.5)

**Table 4:** Descriptive statistics of study participants’ responses to 44 statements in the seven domains of the new scale and the reliability of each domain (N=546).

Statements		Value, mean (SD)	
**Physical domain, seven items (Cronbach alpha=.714)**			
	P1. I feel rested when I wake up in the morning.		3.60 (1.54)
	P2. Each week, I exercise moderately for at least 30 minutes (for instance, walking briskly, bicycling slower than 10 miles per hour, playing tennis, and ballroom dancing).		3.14 (1.13)
	P3. Because of my health status, I am physically able to exercise as much as I would like to.		2.60 (1.63)
	P4. I usually have enough energy for everyday activities.		2.52 (1.32)
	P5. My chronic pain level is (0=no pain, 10=most severe pain ever).		1.23 (1.46)
	P6. My appetite has been good recently.		2.15 (1.05)
	PO. My overall physical health is (1=excellent, 5=terrible).		2.09 (0.69)
**Mental domain, seven items (Cronbach alpha=.860)**			
	M1. I am generally satisfied with my quality of life.		2.44 (1.21)
	M2. I am generally self-accepting.		2.57 (1.29)
	M3. I feel hopeful about the future.		2.46 (1.25)
	M4. I feel that I have control over my emotions.		2.40 (1.05)
	M5. I believe that life is what you make it.		2.31 (1.21)
	M6. I am open to new opportunities if my first plan does not work out.		2.03 (0.79)
	MO. My overall mental health is (1=excellent, 5=terrible).		2.05 (0.78)
**Social domain, six items (Cronbach alpha=.781)**			
	S1. I am living in a safe community.		1.82 (0.88)
	S2. When something good happens to me, I share the experience with my family and/or friends.		1.90 (0.92)
	S3. I am satisfied with my ability to meet the needs of people who depend on me.		2.12 (0.98)
	S4. I am satisfied with my current level of social activities.		2.84 (1.38)
	S5. I have people in my life who care about me.		1.56 (0.78)
	SO. My overall social wellness is (1=excellent, 5=terrible).		2.02 (0.77)
**Financial domain, five items (Cronbach alpha=.856)**			
	F1. If I incur an unexpected above average expense, I would still be stable financially.		3.03 (1.84)
	F2. I have someone to help with my financial affairs, if needed.		2.95 (1.77)
	F3. I am saving for retirement and for emergencies.		2.28 (1.46)
	F4. My income is adequate for my current needs.		3.21 (1.79)
	FO. My overall financial wellness is (1=excellent, 5=terrible).		2.39 (0.93)
**Spiritual domain, six items (Cronbach alpha=.892)**			
	SP1. I feel that my life is meaningful.		2.21 (1.11)
	SP2. I feel inner and/or spiritual strength in difficult times.		2.69 (1.38)
	SP3. I have a sense of direction for my life.		2.45 (1.19)
	SP4. I know what is really important in my life.		2.01 (0.98)
	SP5. My personal beliefs (religious or not) help me to cope with difficulties in life.		2.32 (1.11)
	SPO. My overall spiritual wellness is (1=excellent, 5=terrible).		2.14 (0.79)
**Occupational domain, seven items (Cronbach alpha=.844)**			
	O1. I feel I have input on deciding how my job gets done.		2.52 (1.37)
	O2. I am satisfied with the amount of time required by my job duties.		2.86 (1.49)
	O3. My employer provides me many career development opportunities.		3.06 (1.63)
	O4. I feel comfortable working with my colleagues.		2.21 (1.22)
	O5. My work and life are well-balanced.		2.97 (1.50)
	O6. My job security is high.		2.90 (1.43)
	OO. My overall occupational wellness is (1=excellent, 5=terrible).		2.22 (0.80)
**Intellectual domain, five items (Cronbach alpha=.828)**			
	I1. I am satisfied with the quality of my work.		2.15 (0.99)
	I2. I am aware of my intellectual strengths.		1.92 (0.80)
	I3. I can rely upon my talents and skills to handle unexpected situations.		1.89 (0.75)
	I4. I am satisfied with my ability to make decisions.		2.07 (0.90)
	IO. My overall intellectual wellness is (1=excellent, 5=terrible).		1.78 (0.60)
WO. My overall well-being is (1=excellent, 5=terrible).^a^		1.99 (0.64)	

^a^The statement is for self-assessment of overall wellness. It is in the scale but does not belong to any domain.

**Table 5:** Rotated factor loadings of the exploratory factor analysis with 44 statements from 546 study participants.

Statement ID	Factor 1	Factor 2	Factor 3	Factor 4	Factor 5	Factor 6	Factor 7
P1	0.485^a^	0.351	—	—	—	—	—
P2	0.534^a^	—	—	—	—	—	—
P3	0.758^a^	—	—	—	—	—	—
P4	0.609^a^	—	—	—	—	—	—
P5	0.610^a^	—	—	—	—	—	—
P6	0.170	0.301	—	—	—	—	—
PO	0.621^a^	—	—	—	—	—	—
M1	—^b^	0.447^a^	—	—	—	—	—
M2	—	0.655^a^	—	—	—	—	—
M3	—	0.376^a^	—	—	0.351	—	—
M4	—	0.632^a^	—	—	—	—	—
M5	—	0.488^a^	—	—	—	—	—
M6	—	0.413^a^	—	—	—	—	0.321
MO	—	0.528^a^	—	—	—	—	—
S1	—	—	0.339^a^	—	—	—	—
S2	—	—	0.612^a^	—	—	—	—
S3	—	—	0.574^a^	—	—	—	—
S4	—	0.363	0.590^a^	—	—	—	—
S5	—	—	0.555^a^	—	—	—	—
SO	—	—	0.625^a^	—	—	—	—
F1	—	—	—	0.887^a^	—	—	—
F2	—	—	—	0.683^a^	—	—	—
F3	—	—	—	0.708^a^	—	—	—
F4	—	—	—	0.829^a^	—	—	—
FO	—	—	—	0.891^a^	—	—	—
SP1	—	—	—	—	0.563^a^	—	—
SP2	—	—	—	—	0.820^a^	—	—
SP3	—	—	—	—	0.640^a^	—	—
SP4	—	—	—	—	0.604^a^	—	—
SP5	—	—	—	—	0.809^a^	—	—
SPO	—	—	—	—	0.796^a^	—	—
O1	—	—	—	—	—	−0.709^a^	—
O2	—	—	—	—	—	−0.759^a^	—
O3	—	—	—	—	—	−0.730^a^	—
O4	—	—	—	—	—	−0.676^a^	—
O5	—	—	—	—	—	−0.686^a^	—
O6	—	—	—	—	—	−0.578^a^	—
OO	—	—	—	—	—	−0.784^a^	—
I1	—	—	—	—	—	−0.345	0.408^a^
I2	—	—	—	—	—	—	0.860^a^
I3	—	—	—	—	—	—	0.869^a^
I4	—	—	—	—	—	—	0.768^a^
IO	—	—	—	—	—	—	0.839^a^
WO	0.156	0.288	0.170	0.085	0.173	−0.205	0.225

^a^Selected domain.

^b^Factor loadings <0.32.

There are several statements with high loading factors in more than one domain. For instance, P1 is about sleep quality, which is related to both physical and mental wellness. In these cases, both factor loading and Cronbach alpha were used to determine which domain is more appropriate for the statements. Typically, the domain with the higher factor loading and higher Cronbach alpha was chosen.

The confirmatory factor analysis assessed the fit of the seven-factor structure using the responses from the 546 study participants. For this seven-factor two-layer model, the comparative fit index (CFI) was 0.866, Tucker-Lewis index (TLI) was 0.859, and the root mean square error of approximation (RMSEA) was 0.058, suggesting adequate model fit.

### Comments

Some study participants provided brief comments after providing responses in the well-being scale. Some of these comments were specific to the university and are not shown here. Others were more generic and may be applicable to other places as well. These comments are presented below.

I also have a second job which affects the answers given here.

Participant #42 in Q3

Occupational distress mostly from direct supervisor.

Participant #394 in Q3

Currently pregnant, so my physical well-being is not what it should be.

Participant #547 in Q3

Great survey; easy to understand and take!

Participant #646 in Q3

Not a big fan of spiritual wellness. I know this survey says this could be religious or not, but isn't there another word without a religious connotation?

Participant #648 in Q3

## Discussion

### Principal Findings

The goal of this study was to develop a new comprehensive well-being scale for people in the university environment and evaluate its reliability and validity. We used a combined method (traditional survey design method and crowdsourcing) to create a new well-being scale for people in the university environment. This is a user-centered approach since the target population is involved in all the stages of scale development. The benefits of this combined method and user-centered approach were that findings were incorporated from previous well-being scale development studies and ideas and opinions were gathered from a large number of people in the target population. The obtained scale was shown to be highly reliable (Cronbach alpha of the scale was .933). A summary of the uniqueness of this study is given below.

First, the candidates for domains, subdomains, and scale statements were collected from a large number of existing well-being and quality of life scales identified by several recent review studies [6-8]. This provided a solid foundation for the validity and reliability of the new scale. We also had extensive experience in creating and evaluating scales in previous studies [7,52-61]. Second, in the three steps involving users, a total of 1,231 study participants from the target population contributed their ideas to the development of this scale at different stages. This made it possible to fully incorporate their needs and ideas into the new scale. This is typically not done in the traditional scale development approach. Third, the combined method generated a highly reliable new scale, and this result demonstrates that our approach is feasible for scale development.

In the past, scales were typically created by experts according to their experience and the literature. The target populations were only involved in the last step for the final evaluation. The quality of the scale was strongly determined by the knowledge of the experts in the field and their understanding of the target population. The application of crowdsourcing in this project reduced this dependence.

Crowdsourcing has been used in many previous studies [37-45,62-65]; however, the role of study participants was mainly limited to providing responses to already existing questionnaires, instead of being involved in all stages of the questionnaire development and evaluation. In this study, guided by the user-centered approach, samples of the target population were involved in all stages of questionnaire development and evaluation. They provided invaluable ideas for building this highly reliable well-being scale.

### Comparison With Prior Work

There are many other well-being scales. However, none of them were specifically designed for people in the university environment. Additionally, because this environment includes people who have different ages and different roles, the typical well-being scales for the workplace do not apply well [66-69], especially for students. This study used the findings from other well-being scale development studies and adopted a new approach to building a scale for people in the university environment. This new scale is considered better than other generic or employee well-being scales for more precise well-being assessment of this particular population.

### Limitations

A scoring system for this scale has not been established yet, and thus, it is not feasible to compare the well-being outcomes from this scale with those from other existing scales. In the next step, we will develop a scoring system for the scale and compare the obtained scores with the results from other well-established scales domain by domain. We will perform another study to evaluate the relationship among the domains and determine the weight these domains should have in the overall well-being measure. For this purpose, we are currently creating a website that allows people in the university environment to complete multiple well-being scales online, including this new scale and several other scales. The obtained data will be used for comparison and further analysis.

This study included a large number of staff members (n=867) but a relatively smaller number of students (n=135). The number of participants in each category was sufficient to obtain study results. However, since the number of study participants in the three categories was not well balanced, the results may be biased to some extent. It may be necessary to increase focus on these populations by designing well-being scales for people in each category (ie, one scale for staff, one for faculty, and one for students).

### Conclusions

By using a combined approach (a traditional scale development method and crowdsourcing for idea collection at multiple stages of scale development), a highly reliable and comprehensive well-being scale was created for people in the university environment. This scale may be used for reliable well-being assessment in the population of this environment. The results of the well-being assessment may be used to guide the design of well-being improvement interventions.

## Appendix

Multimedia Appendix 1 Other subdomains mentioned by study participants in Q1.

## Abbreviations

Pitt: University of Pittsburgh
PROMIS: Patient Reported Outcomes Measurement Information System
