# Supplementary material for: Development and Validation of a Comprehensive Well-Being Scale for People in the University Environment (Pitt Wellness Scale) Using a Crowdsourcing Approach: Cross-Sectional Study
Source: J Med Internet Res. 2020 Apr 29;22(4):e15075. doi: 10.2196/15075 (PMC7221649; doi:10.2196/15075)
Supplement: Multimedia Appendix 1 [file jmir_v22i4e15075_app1.docx]

**Table S1**. Other subdomains mentioned by study participants in Q1.

| **Domain** | **Additional Subdomains** |
| --- | --- |
| Physical | disability, flexibility, substance use, sexual activity, pain |
| Emotional | chronic disease of family member, fairness, love, healthy attitude toward food |
| Social | ability to be self in social situations, community belonging, identifying interests and developing hobbies, long-term trusted relationships, social acceptance, social support, work and home environment free from abuse/violence |
| Occupational | accountability for job responsibilities, autonomy in job, constructive performance feedback, nurturing work environment, defined career path, effective communication in the workplace, equality, ethical leadership, fair treatment from employer, fair market salary, feeling valued at work, being shown respect, mental stimulation from job, mentorship, physical safety and health at work, reasonable supervisor expectations, responsive workplace, satisfaction with coworkers, support from supervisor, work-life balance |
| Financial | debt management, finances literacy, financial security, financial stability, opportunities to improve financial status/stability, retirement plan |
| Spiritual | ability to have spirituality without externalization, ability to understand being present, empathy skills, ability to understand others’ spiritual well-being and be respected for own, engagement with meaningful activities, knowledge of and access to resources to answer spiritual questions, ethical/moral development, freedom to express religious beliefs in the workplace without fear of retaliation, personal value and fulfillment |
| Intellectual | ability to learn, cognition, critical thinking, curiosity, engagement, knowledge of and access to life-long learning activities, mentorship, self-motivated on-going education, open-mindedness, openness to new ideas, opportunity to be exposed to diverse lifestyles, work environment that encourages acquisition of new knowledge for everyone |
